# Effects of germicidal far-UVC on ozone and particulate matter in a conference room

**DOI:** 10.1371/journal.pone.0328224

**Published:** 2025-08-11

**Authors:** Farideh Hosseini Narouei, Zifeng Tang, Shiqi Ian Wang, Raabia H. Hashmi, David Welch, Sandhya Sethuraman, David J. Brenner, V. Faye McNeill

**Affiliations:** 1 Department of Chemical Engineering, Columbia University, New York, New York, United States of America; 2 Center for Radiological Research, Columbia University Irving Medical Center, New York, New York, United States of America; 3 Climate School, Columbia University, New York, New York, United States of America; 4 Department of Earth and Environmental Sciences, Columbia University, New York, New York, United States of America; SKUMS: Shahrekord University of Medical Science, ISLAMIC REPUBLIC OF IRAN

## Abstract

The application of 222 nm light from KrCl excimer lamps (GUV222 or far-UVC) is a promising approach to reduce the indoor transmission of airborne pathogens, including the SARS-CoV-2 virus. GUV222 inactivates airborne pathogens and is believed to be relatively safe for human skin and eye exposure. However, UV light initiates photochemical reactions which may negatively impact indoor air quality. We conducted a series of experiments to assess the formation of ozone (${\text{O}}_{3}$), and resulting formation of secondary organic aerosols (SOA), induced by commercial far-UVC devices in an office environment (small conference room) with an air exchange rate of $1.3\;{\text{h}}^{-1}$. We studied scenarios with a single far-UVC lamp, corresponding to the manufacturer’s recommendations for disinfection of a space that size, and with four far-UVC lamps, to test conditions of greater far-UVC fluence. The single lamp did not significantly impact ${\text{O}}_{3}$ or fine particulate matter levels in the room. Consistent with previous studies in the literature, the higher far-UVC fluences lead to increases in ${\text{O}}_{3}$ of 5 to 10 ppb above background, and minor increases in particulate matter (16% ± 10 % increase in particle number count). The use of far-UVC at minimum intensities required for disinfection, and in conjunction with adequate ventilation rates (e.g. ANSI/ASHRAE recommendations), may allow the reduction of airborne pathogen levels while minimizing the formation of air pollutants in furnished indoor environments.

## Introduction

The dominant pathway of transmission for respiratory viruses, including SARS-CoV-2, influenza, and respiratory syncytial virus, is airborne (aerosol) transmission [1,2]. In this scenario, an infected person emits respiratory aerosols containing pathogens which may be inhaled by others, leading to infection. Indoor gatherings, especially in crowded and poorly ventilated settings, pose a higher risk of transmission. Engineering solutions, including adequate ventilation and air filtration, can reduce transmission risk [3,4].

Germicidal ultraviolet (GUV) light has been proposed as another promising tool for reducing the risk of airborne virus transmission indoors. UV light is well-known to inactivate or kill bacteria and viruses on surfaces and in aerosols [5–11], but drawbacks include the potentially harmful impacts of UV rays on human skin and eyes. The use of light in the far-UVC range (200-235 nm) minimizes these negative impacts. Far-UVC is effective at inactivating or killing microorganisms, including bacteria, viruses, and fungi [12–17]. Research indicates that 222 nm light from KrCl excimer lamps (far-UVC) has minimal adverse effects on the skin and eyes, attributable to a limited ability for the radiation to penetrate deeply into biological materials [12,18–20]. Due to its ability to inactivate airborne pathogens and relative safety, far-UVC has been recommended for use indoors to inactivate pathogens, including SARS-CoV-2 [11].

Besides direct damage to skin and eyes, another potential drawback of GUV is negative impacts on indoor air quality due to photochemistry. Oxygen photodissociates in the presence of high energy light (wavelengths shorter than 242 nm):

$$O_{2}+\text{h}\nu \to O+O$$ (R1)

The atomic oxygen generated reacts with ${\text{O}}_{2}$ to yield ozone (${\text{O}}_{3}$).

$$O+O_{2}+M\to O_{3}+M$$ (R2)

Acute and long-term exposure to elevated levels of ${\text{O}}_{3}$, a respiratory irritant and strong oxidant, is harmful to human health [21]. ${\text{O}}_{3}$ also contributes to fine particulate matter levels by oxidizing volatile organic compounds (VOCs) to form semivolatile or water-soluble products which may partition to the particle phase, a process known as secondary organic aerosol (SOA) formation. Terpene compounds associated with scent products in indoor environments, such as limonene, are particularly reactive towards ${\text{O}}_{3}$ and have high SOA formation potential due to their tendency to form semivolatile or water-soluble products upon oxidation [22]. For this reason, studies of the impact of GUV on indoor air quality have included measurements of ozone formation [25–31] and SOA formation [25,26,30,31].

While ozone formation chemistry has been studied extensively for broad spectrum solar light in the outdoor context [23,24], the effects of far-UVC indoors are less well-known. Recent numerical modeling [25,26] and laboratory [26–30] studies show that 222 nm far-UVC can increase ozone levels [25–30] and SOA formation [25,26,30] . The laboratory studies were mostly performed in reactor systems with low or no ventilation. These systems differ in important ways from an indoor office environment, which is ventilated and has various reactive surfaces which act as sinks for ozone [27,32] . Variations in room volume and geometry, surface material reactivity, and ventilation rate could influence both ozone formation and decay, and particulate matter behavior. Peng and coworkers observed ${\text{O}}_{3}$ production and decay from the use of far-UVC (room averaged rate = 2.0 *μ*W cm^−2^) in a small, low-ventilation (0.62-0.96 h^−1^) office setting [27]. They found steady-state production of around 6.5 ppb ${\text{O}}_{3}$, with an inferred ${\text{O}}_{3}$ deposition rate of 0.5-2.3 h^−1^.

Here, we report the impact of commercial far-UVC lamps with a range of intensities on ozone and submicron aerosol particle levels in an indoor office environment (small conference room).

## Materials and methods

### Room setup

The study was conducted in a small conference room (4.62m × 2.74m, 3.30m ceiling height) within an office suite in a 19-story academic building at Columbia University’s Morningside Heights campus in New York, NY, USA from March 2023 to January 2024. The building, constructed in 1961, has a centralized mechanical heating, ventilation, and air conditioning system. We refer to this room as “Room A.” Room A has no window, and is connected via a door to a large, open plan lounge area in the office suite. This lounge space was used for baseline measurements. The door between Room A and the lounge was closed during experiments. The air exchange rate in Room A was measured using the ${\text{CO}}_{2}$ tracer method [33] with portable ${\text{CO}}_{2}$ sensors (Aranet4) to be 1.3 h^−1^ (S1 Fig). Room A and the lounge are carpeted. Room A is equipped with a conference table, a small wooden desk, and chairs. It also has room lighting and a wall-mounted computer monitor. Within the experimental duration none of the electronics were turned on.

### Far-UVC source

During the study, the room was equipped with either one or four commercial fixtures to generate far-UVC radiation. Tests with a single fixture used a Lumenlabs Lumenizer 300 (Lumenlabs, Shanghai, China) and tests with four fixtures used Lumenlabs Zone devices. Each of these fixtures contains three optically filtered KrCl bulbs with a peak emission wavelength of 222 nm. The optical output of a Lumenizer fixture is 55 mW and the optical output of a single Zone fixture is 190 mW. According to the manufacturer, each Lumenizer 300 fixture is capable of disinfecting a 4m x 4m room; therefore, a single Lumenizer 300 unit would be normally deployed for a room the size of Room A in this study. That is to say, the single lamp tests represent “typical” operating conditions for this room and the four-lamp tests were performed to test a higher intensity condition. Lamps were placed on the floor and oriented towards the ceiling for all tests; this placement is unconventional and not recommended for permanent installation, but it allowed for flexibility during temporary deployment for these tests. This positioning may result in different irradiance distribution and air mixing compared to ceiling-mounted installations. A model of the small conference room including the far-UVC fixtures was generated using Visual Lighting software with the GUV package (Acuity Brands, Atlanta, GA) (S2 Fig, S3 Fig). The model was also used to compute the average and maximum horizontal irradiance at 1.8 m down from the ceiling for each installation. Since the lamp arrangement is flipped in the room from a typical ceiling installation, measuring 1.8 m down from the ceiling represents the measurement at 1.8 m above floor height which is typically used to evaluate installations for safety exposure limits as recommended in the ANSI/IES RP-27.1–22 standard. The model for the single lamp installation yielded an average irradiance of 0.2 *μ*W cm^−2^ and a maximum irradiance of 0.9 *μ*W cm^−2^ at 1.8 m height. The model for the four lamp installation yielded an average irradiance of 4.1 *μ*W cm^−2^ and a maximum irradiance of 9.0 *μ*W cm^−2^ at 1.8 m height. For context, the 8-hour Threshold Limit Values recommended by ACGIH for the eye and skin for 222 nm exposure are 160 mJ cm^−2^ and 480 mJ cm^−2^, which equate to average irradiance values of 5.5 *μ*W cm^−2^ and 16.6 *μ*W cm^−2^, respectively [34]. The 8-hour exposure limit recommended by the ICNIRP for 222 nm exposure is 23 mJ cm^−2^, which equates to an average irradiance of 0.8 *μ*W cm^−2^ [35]. This condition is not recommended for occupied spaces and was used here to explore upper-bound chemical effects in a range of conditions similar to those used in previous studies in the literature.

### Ozone and particle measurements

Ozone monitors (2B Technologies, Model 202 Series #2544) were used for ozone measurement inside the conference room and outside in a lounge area where the far-UVC lamps were not present, to obtain a background signal. The concentration was recorded for approximately 8 hours during the daytime at 10-second intervals, measured in units of ppb, and an hourly average reading was computed.

The particle size distribution in the 11.1nm-1.1*μ*m range was monitored during experiments using a scanning mobility particle sizer (SMPS) (Grimm Technologies). The SMPS scan frequency was 7 minutes. Some experiments were performed with only ozone or SMPS measurement.

### Experimental design

The two ozone monitors and/or the SMPS were turned on at the start of the experiments. Background signal was obtained. After the background period, the far-UVC source (1 or 4 lamps) was turned on in the conference room and no change was made to the lounge. Ozone and SMPS data were collected with the far-UVC source and no other changes. Each experiment (1 or 4 lamps) was repeated three or more times.

## Results

In summary, experiments performed with a single far-UVC lamp did not result in a significant change in ozone or fine particulate levels compared to background concentrations in Room A and the lounge. When higher lamp intensity was applied, a small but measurable increase in ozone levels was detected, with concentrations rising by up to 10 ppb above background. Additionally, a small increase in particulate matter was observed (16% ± 10% increase in particle number count). Details are provided in the following paragraphs.

### Ozone

**Fig 1:**
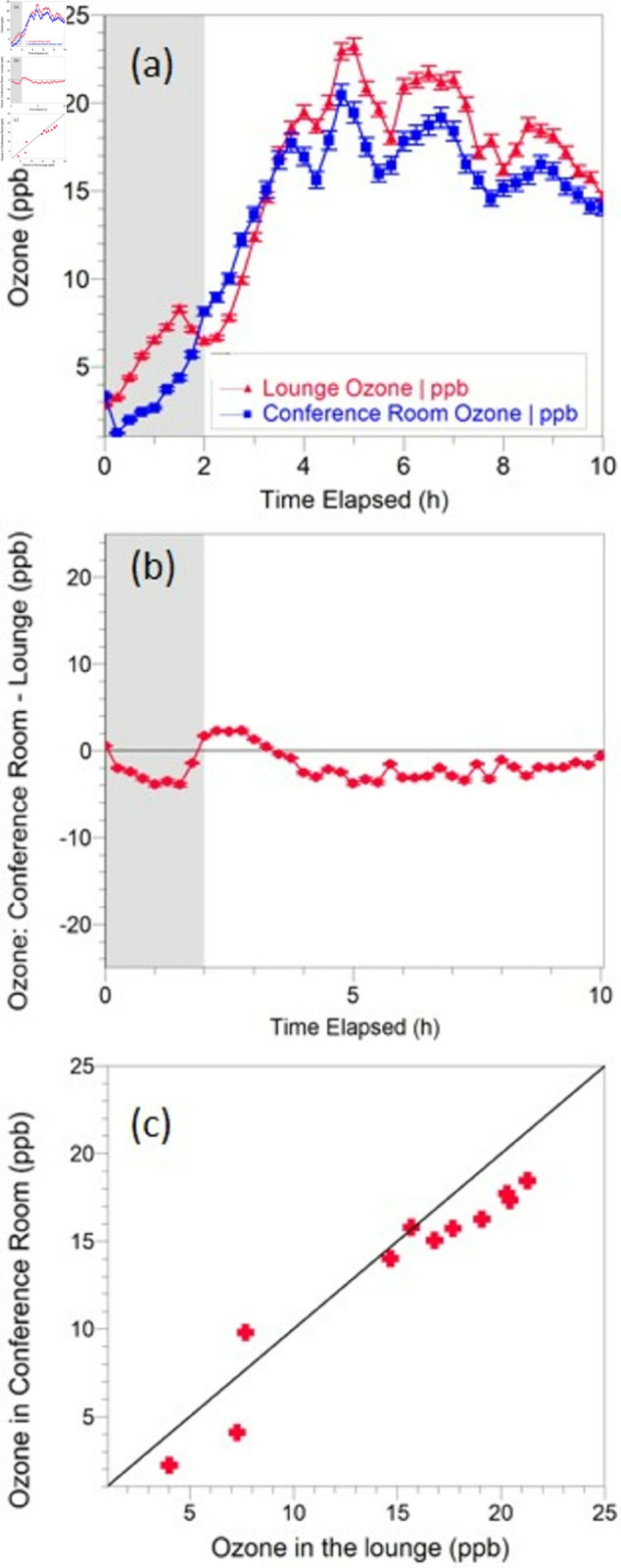
${\text{O}}_{3}$ data for a typical single-lamp experiment. The far-UVC lamp was turned on after 2 hours (The shaded region represents lamp-off period). A: 15 minute averaged ozone readings in the small conference room (Room A) and outside in the lounge. B: Difference plot indicating the difference in ozone level inside Room A and in the lounge. C: Correlation between ozone level in Room A and the lounge.

The 15 minute-averaged measured ozone level in Room A and in the outside lounge for a typical single-lamp experiment is shown in Fig 1 . Background ozone level in the office suite (Room A and lounge area) drifted between 2-20 ppb during the course of the experiments, which lasted up to 10 daytime hours, reaching a peak during midday. This trend suggests that the changes were driven by outdoor ozone concentrations [36] . After 2 hours, a single far-UVC lamp was turned on in the closed Room A. The ozone levels in Room A continued to track the concentrations in the lounge area, and in fact were 2-3 ppb lower, likely due to higher rates of ozone deposition in the small furnished room. The ozone measurements in Room A and the lounge area had a strong linear correlation throughout the 10-hour experiment (ρ=0.96).

**Fig 2:**
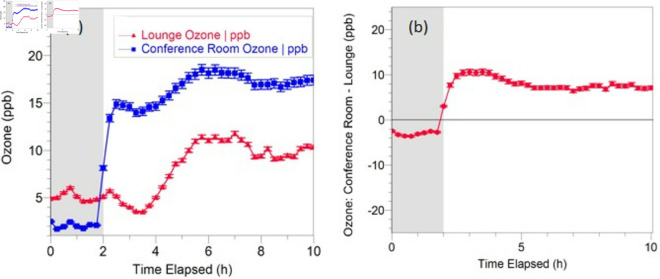
High intensity experiment. Far-UVC lamps were turned on after 2 hours (shaded area represents the lamp-off time interval). A: Ozone level reading in both the small conference room (Room A) and outside in the lounge B: Difference plot indicating the difference in ozone level inside Room A and in the lounge.

The ozone level for a typical higher intensity experiment is shown in Fig 2. Background ozone levels again drifted throughout the 10-hour experiment, with a mid-day maximum (Fig 2(a)). However, when the four far-UVC lamps were turned on at the beginning of hour 3, the ozone level in room A increased to roughly 5-10 ppb above the background (Fig 2(b)). Average results for ${\text{O}}_{3}$ production in single-lamp and higher intensity experiments are shown in Table 1.

**Table 1:** Average ${\text{O}}_{3}$ production results. (Room A – Lounge) difference in ${\text{O}}_{3}$ concentration (ppb), for lamp on and lamp off conditions, for the single lamp and 4 lamp experiments.

Experiment	Lamp off	Lamp on
**1 lamp**	−3.36±0.38	−0.62±0.03
**4 lamps**	−2.56±0.33	6.98±0.24

#### Particle concentration and size distribution.

**Fig 3:**
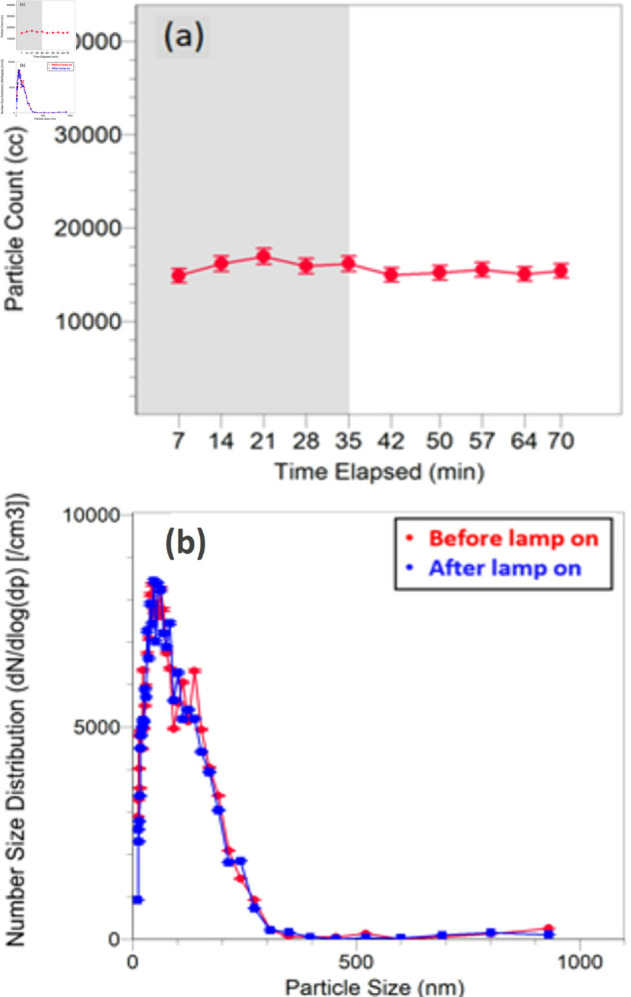
Particle formation results for a typical single-lamp experiment. A: Evolution of particle count during a typical single-lamp experiment. Lamp was turned on after 35 minutes. B: Particle size distribution.

The evolution of particle number and mass concentrations in room A for a single far-UVC lamp is illustrated in Fig 3. Background data were collected for the initial 35 minutes of the experiment. Subsequently, far-UVC was activated and remained on for 35 minutes, during which particle concentrations continued to be monitored. The comparison of particle counts within the entire 70-minute experimental period reveals consistent particle concentrations, with no discernible variation observed when the far-UVC lamp was activated (Fig 3(a)). Particulate matter in Room A typically exhibited a lognormal size distribution with a peak particle size of around 60 nm. The particle size distribution did not change significantly when the far-UVC lamp was turned on (Fig 3(b)).

Averaging data from four single-lamp experiments, we find that the particle number count changes by 142 ± 546 particles cm^−3^ (0.3% ± 3.6% change in number count). Error bars were calculated by propagation of error from the raw experimental data followed by weighted linear least squares averaging. Statistics from only one dataset are available for the high intensity experiment; the observed change was 354 ± 218 particles cm^−3^ (16% ± 10 % change in number count). We note significant variability in the background particle counts, particularly for experiments conducted in Summer 2023. This variability and the existence of only one dataset for the high-intensity 4-lamp particle number analysis limits its statistical interpretation. A two-tailed t-test yielded a p-value of 0.11, suggesting that the change is not statistically significant at the conventional 0.05 threshold.

## Discussion

The far-UVC average fluence used for the lower-intensity (single lamp) experiments in this study was 0.2 *μ*W cm^−2^. According to the manufacturer, one lamp is recommended for disinfection of a room the size of Room A. The modelled average fluence and irradiance values are consistent with conditions observed by Eadie *et al*. in a chamber study to reduce airborne pathogens (*S. aureus*) by 92% or more [16]. Under these conditions, we did not measure significant changes in ozone level or particulate matter in Room A, despite the relatively low ventilation conditions in the room. Note that these experiments represent two lamp configurations in a single room setup, but features of the room such as size, ventilation, and surface material reactivity influence the formation and decay of both ozone and particulate matter.

We note that, if SOA formation is occurring, this could manifest either as new particle formation and growth or as addition of mass to background aerosols. The stable particle number counts and the particle size distribution taken together indicate a lack of evidence for SOA formation under these conditions.

The high intensity experiments employed more far-UVC irradiance than required for disinfection of a room the size of Room A. The increase in ozone of up to 10 ppb above background during these experiments is consistent with the observations of Kalliomäki and coworkers, who used 1.7-1.8 *μ*W cm^−2^ far-UVC in a poorly ventilated hotel quarantine facility room [36], and Peng and coworkers, who used a similar level of far-UVC (2.0 *μ*W cm^−2^) in a small office with low ventilation [27]. Steady state production of 8.6 ppb of ${\text{O}}_{3}$ above baseline was also predicted by simulations of Barber *et al*. for 5 *μ*W cm^−2^ in a room with 1 h^−1^ ACH, assuming an ${\text{O}}_{3}$ deposition rate of 3 h^−1^ [26]. Also consistent with our observations, Kalliomäki did not observe a clear correlation between far-UVC use and submicron particle concentrations with SMPS measurements [36].

A mass balance for ${\text{O}}_{3}$ in Room A can be written as follows:

$$\frac{d[O_{3}]}{dt}=([O_{3,bkd}]-[O_{3}])ACH+R_{O3,gen}-[O_{3}]k_{loss}$$ (1)

That is, the rate of accumulation of ${\text{O}}_{3}$ in Room A is equal to the transport of ${\text{O}}_{3}$ in and out of the room due to the ventilation, plus the ${\text{O}}_{3}$ generation rate from the lamp(s), minus the loss rate due to deposition or other reactive losses. At steady-state,

$$R_{O3,gen}\approx [O_{3}]k_{loss}-([O_{3,bkd}]-[O_{3}])ACH$$ (2)

We can apply this analysis to hours 4-10 of the representative single lamp experiment shown in Fig 1. We take the lounge concentration as [${\text{O}}_{3,bkd}$] and use the measured ACH of 1.3 h^−1^ and 17 ppb as the Room A ${\text{O}}_{3}$ level at steady state (Fig 1. ). There is some uncertainty in the depositional loss rate ${\text{k}}_{\text{loss}}$, with rates for carpeted rooms reported in the literature ranging approximately 0.5-2.3 h^−1^ [27,37]. This range of assumed ${\text{k}}_{\text{loss}}$ corresponds to an approximate ozone generation rate for the Lumenizer 300 of 5.25-36 ppb h^−1^. This is within the range of observations for other GUV lamps in real-world settings [27,31]. We can calculate a hypothetical minimum ${\text{k}}_{loss}$, which would correspond to zero ozone generation, of 0.19 h^−1^. Given the dimensions of Room A, this corresponds to an ozone deposition velocity of 0.53 cm s^−1^, which falls within the range reported for ${\text{O}}_{3}$ deposition to carpeted surfaces [37].

Better ventilation (>3 h^−1^), as recommended by ASHRAE, would further suppress ozone buildup and improve indoor air quality under far-UVC use.

## Conclusion

We have observed the effects of far-UVC on indoor ozone and particulate matter under conditions of real-world application in a small conference room with low ventilation. We find that a single Lumenizer 300 far-UVC lamp, specified by the manufacturer to be sufficient for disinfection of the space, does not result in significant net generation of ${\text{O}}_{3}$ or PM under the conditions of our experiment. Far-UVC may be a valuable component of a multilayer approach to reduce the risk of transmission of respiratory viruses, used in combination with ventilation and other interventions including air filtration, in furnished indoor environments. The smallest possible irradiance necessary for disinfection should be used for the application in order to minimize ozone generation and any possible effects on skin or eyes. Better ventilation than we observed in Room A would further reduce steady state ozone buildup and is recommended for improved indoor air quality in general [4].

## Supporting information

S1 FigData for ventilation characterization of Room A.${\text{CO}}_{2}$ tracer decay experiment was conducted following McNeill *et al*. (2021). Two Aranet4 ${\text{CO}}_{2}$ monitors were placed at opposite ends of the room. ${\text{CO}}_{2}$ (Airgas) was released into the room until the measured concentration of ${\text{CO}}_{2}$ was 1000 ppm. The decay in ${\text{CO}}_{2}$ reading was observed and ACH was derived using an exponential fit to the data (1.3 h^−1^).(TIF)

S2 Fig The visual lighting modeling result for single lamp experiment. (TIF)

S3 Fig The visual lighting modeling result for 4 lamp experiment. (TIF)
