# Supplementary figures and images for: Effects of germicidal far-UVC on ozone and particulate matter in a conference room
Source: PLoS One. 2025 Aug 11;20(8):e0328224. doi: 10.1371/journal.pone.0328224 (PMC12338833; doi:10.1371/journal.pone.0328224)

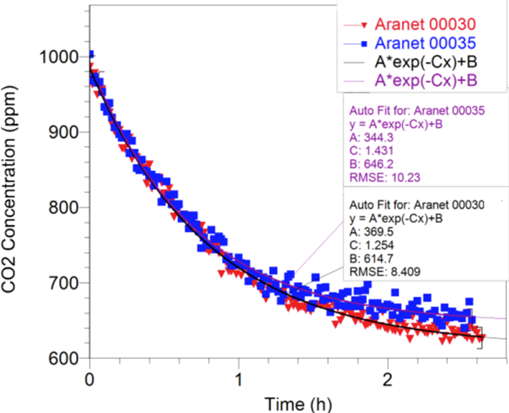

Supplement: S1 Fig — CO2 tracer decay experiment was conducted following McNeill et al. (2021). Two Aranet4 CO2 monitors were placed at opposite ends of the room. CO2 (Airgas) was released into the room until the measured concentration of CO2 was 1000 ppm. The decay in CO2 reading was observed and ACH was derived using an exponential fit to the data (1.3 h−1). (TIF) [file pone.0328224.s001.tif]

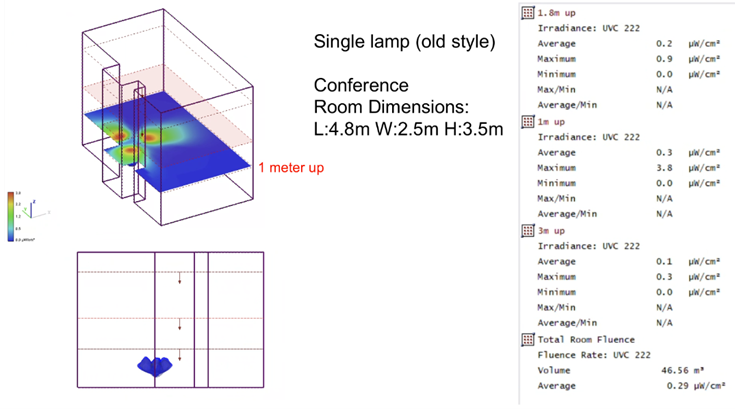

Supplement: S2 Fig — (TIF) [file pone.0328224.s002.tif]

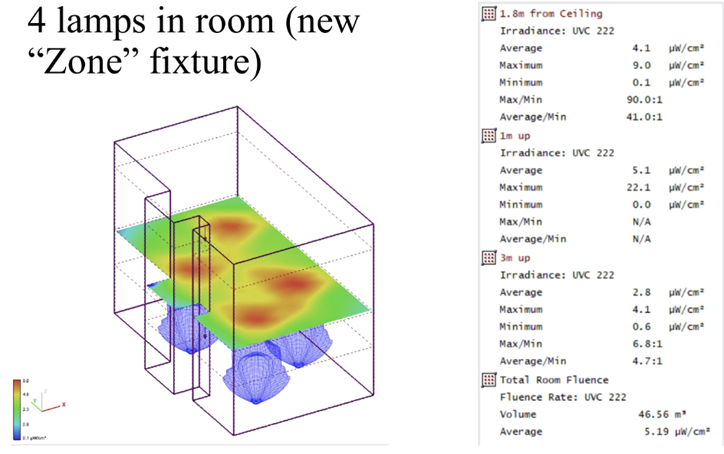

Supplement: S3 Fig — (TIF) [file pone.0328224.s003.tif]
